# The relationship between diabetic ketoacidosis and suicidal or self-injurious behaviour: A systematic review

**DOI:** 10.1016/j.jcte.2023.100325

**Published:** 2023-09-29

**Authors:** Lina Al Alshaikh, Anne M. Doherty

**Affiliations:** a Royal College of Surgeons, Dublin, Ireland; b Department of Psychiatry, University College Dublin, 63 Eccles Street, Dublin 7, Ireland; c Department of Liaison Psychiatry, Mater Misericordiae University Hospital, 63 Eccles Street, Dublin 7, Ireland

**Keywords:** Type 1 diabetes mellitus, Suicide, Self-harm, Diabetic ketoacidosis, Psychiatry

## Abstract

**Background:**

It has been suggested that there may be an association between type 1 diabetes (T1DM) and suicide, with one study reporting a rate 11 times that of the general population The aim of this paper was to investigate the association between Diabetic ketoacidosis (DKA: a life-threatening acute complication of T1DM) and suicidal behaviours in people with T1DM.

**Methods:**

We performed a search of the following databases: PubMed, PsychInfo, and Embase for papers which explored the association between suicidal behaviours and self-harm with DKA in T1DM. We excluded case reports and review papers.

**Results:**

Only three papers explored the relationship between DKA and self-harm. One study found an association between DKA and self-harm in a national cohort of people with type 1 diabetes and schizophrenia. The second found a significant increase in psychiatric admissions for self-harm following an episode of DKA. The third study reported that patients with diabetes and a history of self-harm were at elevated risk of a range of diabetes complications including DKA. These findings indicate an association between DKA and self-harm and support the guidelines in recommending a psychosocial assessment where DKA cannot be explained.

**Conclusions:**

This review suggests that DKA is associated with suicidal or self-injurious behaviours. The small number of studies and the seriousness of this issue highlight the importance of further research on this topic, to improve the evidence base for the identification and treatment of risk of suicidal behaviours in people with T1DM.

## Highlights

• There is an association between type 1 diabetes and suicidal behaviours.
• This preview reported a relationship between DKA and self-harm in type 1 diabetes.
• A psychosocial assessment is required where DKA cannot be explained.

## Introduction

### Diabetic ketoacidosis

Diabetic ketoacidosis (DKA) is a potentially life-threatening acute complication of type 1 diabetes mellitus [1]. It occurs when there is inadequate insulin and is characterised by a triad of hyperglycaemia, the presence of serum ketones and acidosis. Insulin deficiency may be a consequence of either an absolute lack of availability of insulin, or a significant increase in demand such as in the case of infection [2]. It triggers a cascade of endocrine changes including increased release of glucagon, catecholamines and growth hormone, resulting in gluconeogenesis and glycogenolysis which also results in increased blood glucose. Insulin deficiency results in high serum glucose, but also a difficult in intracellular transport of glucose resulting in cellular starvation, and a reliance on fatty acids for energy. This results in lipolysis and the accumulation of fatty acids in serum and the liver, where they are oxidized resulting in the formation of ketone bodies: hydroxybutyrate, acetone and acetoacetate. It triggers a cascade of endocrine changes as a stress-response including increased release of glucagon, catecholamines and growth hormone, resulting in gluconeogenesis and glycogenolysis which also results in increased blood glucose, and the body enters a catabolic state [2]. DKA is often multifactorial and may be triggered by factors such as infection, poor adherence to insulin therapy, pump failure, difficulties at injection/pump sites in addition to newly diagnosed diabetes [3], [4], [5]. Insulin omission is the most common cause of DKA [6].

DKA is a medical emergency and without prompt treatment, DKA can be fatal. DKA is the leading cause of death in people under 50 years with type 1 diabetes, with a Scottish national registry study demonstrating that DKA contributed to 29 % of male deaths and 22 % in women [7]. People with recurrent episodes of DKA have six-year mortality rates of 23.4 %, four-times the mortality rates of those who have only one episode of DKA [8].

DKA has been associated with neuro-cognitive complications in children, and there is evidence that DKA especially at time of diagnosis is associated with poorer neurodevelopmental outcomes including lower IQ [9]. This has been postulated to be associated with the “stress diathesis hypothesis” which suggest that hyperglycaemia may trigger structural and functional changes within the brain itself as well as at the blood–brain barrier, resulting in increased permeability of the blood–brain barrier to potential neurotoxins [10]. This may increase vulnerability to poorer outcomes, especially when experienced at a younger age. A life-threatening complication of DKA is cerebral oedema, which may occur in adults as well as children, which may be due to the inflammatory response especially interleukin-1 [11]. One case has associated DKA with a metabolic encephalopathy, although in the case there were a number of potential contributory factors including a preexisting neurological disorder and substance use [12]. There is one case in the literature of a DKA-induced episode of delirium, in an adolescent [13]. In diabetes more broadly hyperglycemia-related brain injury is more commonly seen in chronic hyperglycaemia and the associated microvascular complications. Its aetiology is complex with neurodegeneration, neuroinflammation, vascular disease, oxidative stress, mitochondrial dysfunction, changes in neurotransmitter action, the accumulation of amyloid β and tau phosphorylation all having been implicated [14].

### Diabetes, diabetic ketoacidosis and mental health

Patients with psychiatric disorders are at increased risk of DKA due to impaired glucose monitoring [15]. Patients who suffer from depression and type 1 diabetes are at a higher risk of DKA [16], [17]. However, there may be greater complexity involved in this relationship, and a recent paper by Garrett et suggested a significant relationship between poor diabetes control resulting in repeated episodes of DKA and attachment difficulties [18].

Roberts et al. reported that people with type 1 diabetes had a significantly increased rate of suicide: 11 times that of the general population [19]. Subsequent studies have confirmed an elevated rate of suicide among people with diabetes, although not to the same magnitude. Roy et al. reported an increased rate (3 to 4 times) of suicides attempts among African-American patients with type 1 diabetes in New Jersey compared with controls without diabetes (13.3 % vs. 3.5 %), and 10 % of patients had ongoing suicidal ideation [20]. This study reported that on multivariate analysis a number of key factors including being female, higher numbers of traumatic events in childhood, depression and alcohol abuse were independently significantly associated with attempted suicide and self-harm in this population. Where there was more than one adverse childhood event, each additional type of childhood trauma was associated with an increased risk of suicide attempts by 50 %, after controlling for other factors. There were higher rates of smoking, alcohol abuse, and drug abuse among those with suicidal behaviours, all of which are markers for poorer outcomes [20].

Since suicidal ideation is often seen in patients who suffer from depression, it is important to explore the link between suicidal behavior and DKA [21], [22].] Moreover, understanding the psychopathology of DKA is crucial as it allows physicians to include mental health treatment when managing DKA. In addition, the more aware physicians are of warning signs of suicide, the more likely they will be able to identify suicidal ideation in patients with DKA.

Diabetes can present a particular challenge in the management of suicidal ideations and behaviours given that insulin, the essential treatment for type one diabetes (and used by many individuals with type 2 diabetes) is potentially lethal in overdose. For example, psychiatrists would generally avoid prescribing medications which can be lethal in overdose, such as tricyclic antidepressants, in patients with a history of suicidal behaviours or current ideations, but in the case of a person with diabetes who requires insulin, this potentially lethal means cannot be easily removed.

### Aims

The aim of this paper is to investigate the relationship between DKA and suicidal or self-injurious behavior and to explore the link between DKA and subsequent suicidal behaviours.

## Methods

This study is a systematic review of the relationship between DKA and suicidal behaviours. It was based on the Preferred Reporting Items for Systematic Review and Meta-Analysis (PRISMA) guidelines for systemic review and *meta*-analysis [23]. We did not require ethical approval as the study involved secondary analysis of anonymised data in the public domain.

To perform a systematic review looking into DKA and suicide, we conducted a search of the following databases: Pubmed, Psychinfo, and Embase to identify relevant papers. We included all articles from the beginning of records until June 2021. We restricted the search to those studies published in peer-reviewed journals. We included original research papers only, and did not include case reports or series, or systematic or narrative reviews.

**Fig. 1:**
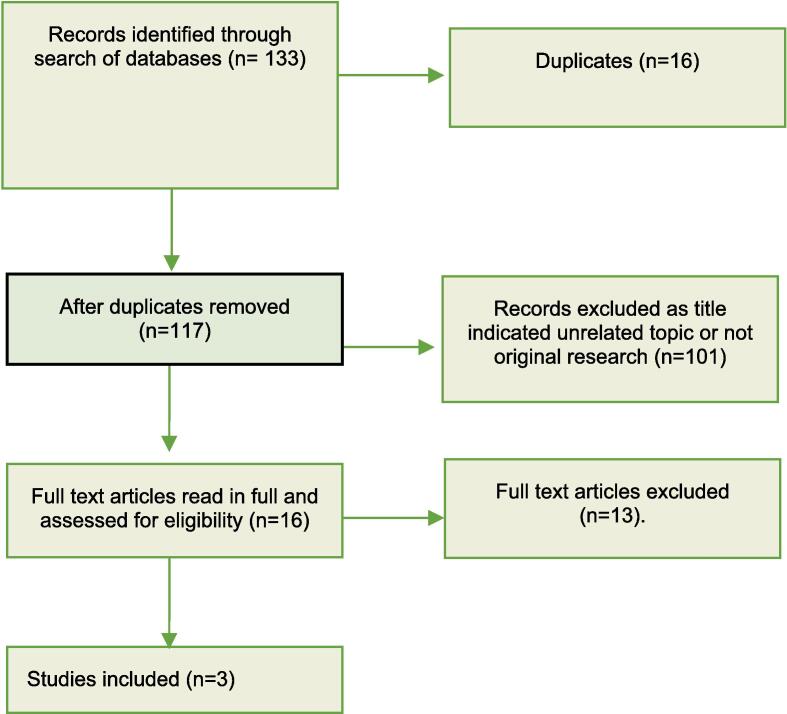
PRISMA flow chart for the selection of studies in the systematic review.

The search terms we used were: (suicid* OR self-harm OR self-injur*) AND (DKA OR Diabetic Ketoacidosis). The search was conducted on June 28, 2021. The studies were examined, as shown in Fig. 1, by two reviewers who, blind to each other, each identified relevant studies by titles and then abstracts. Finally, they examined the full texts and agreed on three papers to be included in this systematic review (Fig. 1).

### Study design and assessment

We assessed the quality of the included papers using the Oxford scale as well as a quality assessment modeled from Pompili et al. [22]. The maximum score was 8, and studies were evaluated according to the criteria below:

I) “Representiveness of the sample to the general population (1point): 0 points not representative; 1 representative
II) Presence of a control group (1 point): 0 points without control group, 1 point with control
III) Number of people in treatment group (1 or 2 points). 0 points if N < 100; 1 point if N < 500; 2 points if N > 500
IV) Duration (years) of follow-up (1 or 2 points). 1 point < 1 year; 2 points > 1 year
V) Data presentation (1 point). 0 points unclear data presentation in the text; 1 point, clear data presentation in the text
VI) Evidence-based measures assessing suicide or suicide attempts (1 point): 0 points absence of evidence-based measures assessing suicide or suicide attempts; 1 point presence of evidence-based measures assessing suicide or suicide attempts.”

## Results

The combined search strategies yielded a total of 133 titles to be screened, and 117 remained after the removal of duplicates (Fig. 1). Following review of title and abstract, 16 remained. These studies were reviewed in full. No further studies were added following a hand-search of the references of the included studies. Of the sixteen studies reviewed in full, this study found only three papers which met the inclusion criteria: these three papers addressed the relationship between DKA and suicidal or self-injurious behaviour (Table 1).

**Table 1:** Description of the included studies.

**Study**	**Study design**	**Sample**	**Follow up**	**Methods**	**DKA as risk factor**	**Conclusions**
Goueskard et al.	Retrospective cohort study	Age: 15–35 Patients with type 1 diabetes and schizophrenia: 341 Patients with type 1 diabetes only: 45,314	Yes	Patients with type 1 diabetes and schizophrenia were identified and compared with patients with type 1 diabetes only. They were assessed for acute diabetes complications, suicide attempts and hospital mortality.	yes	Schizophrenia was associated with increased risk of hospitalization for acute diabetes complications, as well as suicide and hospital mortality. The study also noted that suicide attempts and acute diabetes complications are associated independently of schizophrenia.
Eckert et al.	Cross-sectional case-control study	Age: 8–25 Type 1 diabetes patients (82007) divided into: 1. Type 1 diabetes no self-harm or psychiatric comorbidities = 81840 2. Type 1 diabetes AND self-harm +/- psychiatric comorbidities = 167 3.Control: type 1 diabetes no self-harm OR psychiatric comorbidities = 76050	No – cross sectional and duration of time on database not reported	Diabetes registry was used to identify individuals aged 8–25 with type 1 diabetes. Clinical diabetes-related outcome was compared for patients with self-harm and those without.	Yes	Patients with self-harm had significantly higher HbA1c, higher insulin doses, more DKA events per year, more hospital days per patients and more frequent hospital admissions compared to control group with type 1 diabetes..
Petit et al.	Retrospective cohort study	Age: 18–35 1.Young adults hospitalized for DKA: 1539 2.Young adults not hospitalized for DKA: 14,892	Yes	Examining hospital data for all young adults in France who were hospitalized in 2008 and then examined their subsequent hospitalization for DKA and defined their two sample groups. Psychiatric and medical records were followed up for 10 years.	Yes	Adults admitted to hospital for DKA have an increased risk of future admission following selfharm or suicide attempt. The association between hospitalization for DKA and suicide attempts was strongest in the year following DKA.

Goueslard et al. reported an association between DKA and self-harm in a national cohort of people aged 15–35 years with type 1 diabetes and schizophrenia [24]. This study reported that schizophrenia was associated with an elevated risk of hospital admission for acute diabetes complications, along with higher rates of suicide and of in-hospital mortality. The authors noted that suicide attempts and acute diabetes complications including DKA are associated independently of the diagnosis of schizophrenia.

Erkert et al. reported that young adults aged 8–25 years with diabetes and a history of suicidal or self-injurious behaviour had significantly higher HbA1c and were at elevated risk of a range of diabetes complications including DKA, compared to those without any psychiatric comorbidities [25].

Petit et al. found a significant increase in psychiatric admissions for suicidal or self-injurious behaviour following an episode of DKA [26]. This French population-based study examined the incidence of suicidal behaviours which resulted in psychiatric admission in the years following admission with DKA. The outcomes for these patients were compared with those people with diabetes who were never admitted for DKA. They found that 7 % of patients with type 1 diabetes and a history of DKA were hospitalized during the nine-year follow up period compared to 2.5 % of individuals with type 1 diabetes and no history of DKA. The rate increased to 16.2 % among those patients with type 1 diabetes, previous DKA and a history of mental illness. The rates of self-harm were highest in the first year following the admission for DKA.

The papers were of moderate to good quality. Petite et al. scored 8 on the Quality scale adapted from Pompili, and Goueskard and Eckert scored 5 and 4 respectively (Table 2) [24], [25], [26].

**Table 2:** Quality assessment of the included studies.

**Study**	Quality score*							**Oxford score**
	**a**	**b**	**c**	**d**	**e**	**f**	**TOTAL**	
Goueskard et al.	0	1	1	1	1	1	5	2b
Eckert et al.	0	1	1	1	0	1	4	3b
Petit et al.	1	1	2	2	1	1	8	2b

*Quality scores were calculated using the same criteria as Pompili et al. Quality ratings reported have 8 as a maximum score. StudIes were rated for the quality assessment using the following criteria specified below: a. Representiveness of the sample to the general population (1point): 0 points not representative; 1 representative. b. Presence of a control group (1 point): 0 points without control group, 1 point with control. c. Number of subjects in treatment group (1 or 2 points). 0 points if N<100; 1 point if N<500; 2 points if N>500. d. Duration (years) of follow-up (1 or 2 points). 1 point < 1 year; 2 points > 1 year. e. Data presentation (1 point). 0 points unclear data presentation in the text; 1 point, clear data presentation in the text. f. Evidence-based measures assessing suicide or suicide attempts (1 point): 0 points absence of evidence-based measures assessing suicide or suicide attempts; 1 point presence of evidence-based measures assessing suicide or suicide attempts.

## Discussion

### The relationship between DKA and suicidality in people with T1D

This review found only three studies which explored the relationships between DKA and suicidality in people with T1D. All reported an association between DKA and self-harm or suicidal behaviours. Goueslard et al. reported a relationship between DKA and diabetes complications, suicide, and all-cause mortality in people with schizophrenia [24]. Erkert et al. reported higher rates of DKA among young people with T1D and self-harm [25]. Petit reported an excess of psychiatric admissions following self-harm following an episode of DKA [26].

### Insulin omission as a cause of DKA, a potential link

Insulin omission is the leading cause of DKA [4]. In past decades, patients who presented with recurrent episodes of DKA were often described as having ‘brittle diabetes’, and the difficulties of these patients often confounded the best efforts of clinicians and researchers [27]. Bryden et al. reported that having a psychiatric diagnosis predicted DKA, and conversely, DKA predicted the development of a mental disorder at 10 years [28]. Pelizza and Pupo, in their study which examined patients with recurrent DKA and compared them to a group of patients with stable diabetes, found no difference between the two groups in terms of mental illness, but significantly higher rates of personality disorders in the ‘brittle’ group [29]. Garrett et al.’s review of the literature around ‘brittle’ diabetes noted that clear protocols have developed for the management of recurrent hypoglycemia, but that recurrent DKA has been less researched and therefore there is less evidence for the long-term management of this [30]. In adolescents, Goldston et al. identified that in adolescents were associated with poor diabetes self-management [31], Custal et al. suggested that patients with type 1 diabetes may misuse insulin to deal with difficult emotions, in a similar way that self-harm can be used in other populations to manage difficult emotions and to signal to others that they are in difficulty. Where low levels of motivation to change and/or insulin abuse are suspected in type one diabetes patients, it may be helpful to consider the individual’s personality and role of insulin abuse when determining the appropriate intervention [32]. Boileau et al. recommended that all children with more than two repeated comas within a 3-month period should be admitted to hospital to allow for full evaluation and for multidisciplinary support once it has been discussed secret self-administration of insulin is suspected [33].

### Relationship between diabetes and mental illness

Lofman et al., reported that 81 % of those with type 1 diabetes who died from suicide were previously been hospitalized due to depression, substance abuse or both, in their population based study of completed suicide in Northern Finland [34]. This study examined all cases of death by suicide (n = 2489) over thirteen years Northern Finland. Diabetes was present in 3.1 % of the population who died by suicide: of these 34.6 % had type 1 diabetes. This study found that poisoning was the cause of death in almost half of the people with type 1 diabetes, twice as high as those without diabetes. In half of these cases insulin was the toxic substance consumed. The majority were male and died from a monopharmacy overdose of insulin. Insulin was less frequently used among people with type 2 diabetes who died by suicide, and not at all in people without a diagnosis of diabetes who died by suicide [34]. Pompili et al. conducted a systematic review which reported a high suicide rate among young men with type 1 diabetes, with a peak between the ages of 15 and 29 years. They recommended that physicians should routinely ask their young patients about suicidal thoughts, given the relationship of these thoughts to adherence with the medical treatment [22]. Teenagers with diabetes have high rates of suicidal thoughts, noncompliance with the medical regimen and psychiatric disorder. Adolescents who have a lifetime history of suicidal ideation were found to be more likely to be non-compliant with their medical regimen [29].

### General principles for suicide prevention

In suicide prevention in general populations, means reduction is a key component in reducing the risk of suicide. There is international evidence by restricting access to lethal means, overall rates of death by suicide can be reduced [34]. This was first noted with the reduction of carbon monoxide in coal gas in the UK in the 1960 s resulting in a reduction in suicide, not only by this means, but by any means [35]. When pack sizes of over-the-counter paracetamol were reduced in the UK there was a significant long-term reduction in numbers of deaths due to paracetamol poisoning [36]. Psychiatrists usually avoid prescribing tricyclic antidepressants to people with active suicidal ideation as the risk–benefit ratio inclines towards the risk, and there are alternatives antidepressants with an equivalent effect on depression and suicidality [37]. It is impossible to avoid insulin for patients with type 1 diabetes. In some situations, it may be appropriate to maximise supervision for people assess as being high risk, for example those in who have been admitted to an acute hospital with self-harm. This must be balanced with the need to optimise independence and promote self-management of diabetes. Alongside such measures, it may be useful to have a ‘red flag’ system to identify patients with DKA who require formal psychiatric assessment and treatment [15].

### Implications for clinical care

These findings indicate an association between DKA and suicidal or self-injurious behaviour, and support the guidelines in recommending a psychosocial assessment where DKA cannot be explained [38]. These findings may indicate that an episode of DKA may indicate distress which later manifests as self-harm or suicidal ideation. This indicates that an episode of DKA is a key point for mental health intervention, both in terms of addressing underlying mental health problems which may carry a risk of self-harm and in putting measures in place to avoid further episodes of DKA or other acute complications. There is some evidence that there may be shared inflammatory pathways common to diabetes and depression, and it is likely that by extension, similar mechanisms may well underly the relationship between the severe syndrome of each [39]. Given that there is some promise in the use of mental health treatments for people with recurrent DKA, treatment of co-morbid mood and other mental disorders integrated with diabetes care [40].

### Limitations

Limitations of this research include the small number of studies which were conducted which examined the relationship between DKA and suicidal acts.

### Future directions

There is a real need for further research in this area to inform out knowledge of how DKA and self-harm relate to one another in people with type 1 diabetes to develop targeted interventions. There is a gap in the literature to inform our understanding of common physiological pathways which may help to explain the relationship between DKA and suicidality in T1D, There is some evidence that there may be shared inflammatory pathways common to diabetes and depression, and it is likely that by extension, similar mechanisms may well underly the relationship between the severe syndrome of each [39]. Given that there is some promise in the use of mental health treatments for people with recurrent DKA, treatment of co-morbid mood and other mental disorders integrated with diabetes care [40].

### In conclusion

This review suggests that DKA may associated with increased rates of suicidal and self-injurious behaviours, including in patients with a diagnosis of severe mental illness. There is a need to identify the variables which are associated with self-harm and suicide in people with type 1 diabetes to develop targeted interventions.

## Funding

This research did not receive any specific grant from funding agencies in the public, commercial, or not-for-profit sectors.

## Competing interests

The authors report no proprietary or commercial interest in any product mentioned or concept discussed in this article.

## Declaration of Competing Interest

The authors declare that they have no known competing financial interests or personal relationships that could have appeared to influence the work reported in this paper.
